# Gastroparesis: A Multidisciplinary Approach to Management

**DOI:** 10.7759/cureus.21295

**Published:** 2022-01-16

**Authors:** Stella-maris Chinma Egboh, Sarah Abere

**Affiliations:** 1 Internal Medicine/Gastroenterology, Federal Medical Centre, Yenagoa, NGA; 2 Internal Medicine, Rivers State University Teaching Hospital, Port Harcourt, NGA

**Keywords:** multidisciplinary, interstitial cells of cajal, gastric emptying, management, gastroparesis

## Abstract

Gastroparesis is a neuromuscular disorder whose hallmark is delayed gastric emptying. It is a global challenge to the healthcare system because of poor treatment satisfaction for both the patients and clinicians, eventually leading to a reduction in the quality of life, with antecedent anxiety and depression. Although it is multifactorial in origin, diabetic, idiopathic, and drug-induced gastroparesis are the major risk factors. Disrupted interstitial cells of Cajal (ICC) and gastric dysrhythmia are pivotal to the pathogenesis, with most of the investigations targeted toward assessing gastric emptying and accommodation usually affected by distorted ICC and other neural networks. The treatment challenges can be overcome by a multidisciplinary approach involving gastroenterologists, gastrointestinal surgeons, biomedical engineers, nutritionists, psychologists, nurses, radionuclide radiologists, pharmacists, and family physicians. The exploration of the fundamental physiological processes underlying gastroparesis with the use of biomechanical materials should be given more attention by biomedical engineers to integrate innovative engineering with medicine for solving complex medical issues.

## Introduction and background

Gastroparesis is conventionally defined by delayed gastric emptying without mechanical obstruction [1-3]. It is a common neuromuscular disorder associated with abnormal gastric motility, visceral hypersensitivity, and mucosal inflammation [1,4]. It causes a huge economic burden and has a substantially detrimental effect on the patients’ quality of life [2,5]. There has been a renewed interest in the pathophysiological mechanisms and targeted therapeutic approaches of gastroparesis [2]. Describing the global epidemiology of gastroparesis is challenging because of symptom overlap with functional dyspepsia [6]. The true prevalence of gastroparesis is unknown because a majority of patients do not present to gastroenterologists [7].

In a retrospective study in the United Kingdom, Ye et al. [7] reported a prevalence of 13.8 per 100,000 individuals in 2016, and a standardized incidence of 1.5 per 100,000 person-years in 2004, which increased to 1.9 per 100,000 person-years in 2016 [7]. In the United States, a prevalence of 0.16% was reported in a population-based study [8]. The prevalence was higher among females in all subgroups, accounting for 66.1% of all gastroparesis patients in the same study [8]. This finding is also similar to that by Camilleri et al. [9] who also reported a female preponderance (84%). In their study, a majority of patients (77%) were non-Hispanic whites, 12% were Hispanic, 9% were non-Hispanic black, and 3% were non-Hispanic other racial groups [9]. In a study by Friedenberg et al. [10], the prevalence of gastroparesis was significantly higher among non-white patients compared to white patients (55% vs. 19%). In their study, non-white patients had the highest severity of symptoms, poorer quality of life, and increased utilization of healthcare resources [10]. This review aims to describe the advances in the management of gastroparesis and the complementary role of different specialties.

## Review

Overview of the pathophysiology of gastroparesis

The current understanding of the pathophysiology of gastroparesis is an evolving field in medical practice [11]. Gastric emptying that defines gastroparesis depends on gastric accommodation and motility. Gastric accommodation is a postprandial reflex that decreases gastric wall tension predominantly in the proximal stomach, providing a reservoir for an ingested meal [12]. Both gastric emptying and accommodation are controlled by excitatory cholinergic innervations mediated by the vagus nerve [1,13] as well as inhibitory innervations mediated by the nitrergic nerves which synthesize nitric oxide [14]. The inhibitory innervation is responsible for the relaxation of the pylorus [6,15]. The interstitial cells of Cajal (ICC) that serve as a pacemaker control the functions of these gastric innervations [15]. The ICC generates bioelectrical slow waves that propagate radially toward the distal antrum at a speed of approximately 3 mm/second [13,16].

At the pylorus, the continuity of the ICC is disrupted which serves as a separation from the duodenal slow-wave pattern with a frequency of approximately 12 cycles/minute [17,18]. Although gastroparesis is multifactorial in origin, distortion of the ICC neural connections and gastric dysrhythmias are critical in its pathophysiological mechanism [19]. O'Grady et al. reported that the mean ICC count was significantly reduced with an antecedent risk of conduction abnormalities [17,19]. The aberrant conduction pathway is currently gaining attention in medical literature as a novel pathway for postsurgical gastric dysfunction [17]. These potentials are propagated in a retrograde fashion from the antrum toward the body of the stomach impairing normal gas­tric propulsion [16,20].

Emerging animal studies in gastroparesis have suggested the possible implication of innate immunity [21,22]. This has energized the targeting of cellular and molecular dysfunction in gastroparesis. The neural pathway also has some modulations by the endocrine system, often referred to as the neurohormonal pathway. Ghrelin produced by endocrine cells in the stomach has structural similarities with motilin. It depolarizes the pacemaker potentials of ICC, thereby stimulating gastrointestinal (GI) motility [23]. The role of the platelet‐derived growth factor receptor α positive (PDGFRα+) cells in the pathogenesis of gastroparesis has been suggested. In the stomach, they serve as a control to gastric motility by transducing input signals from the enteric nervous system [5].

It has been postulated that ICC, PDGFRα+ cells, and smooth muscle cells form a syncytium. This controls intracellular voltage-gated calcium (Ca^2+^) in a cyclical manner leading to high-amplitude contractions [24]. A positive correlation exists between velocity and extracellular amplitude which is physiologically necessary for the normal gastric motility regulation [25]. In the antrum, there is a breakdown of solid food into smaller pieces through rhythmic contractions. These food particles are then emptied into the duodenum. The rate of gastric emptying is highly controlled to regulate food entry into the duodenum for optimal absorption [13].

Risk factors for gastroparesis

**Figure 1:**
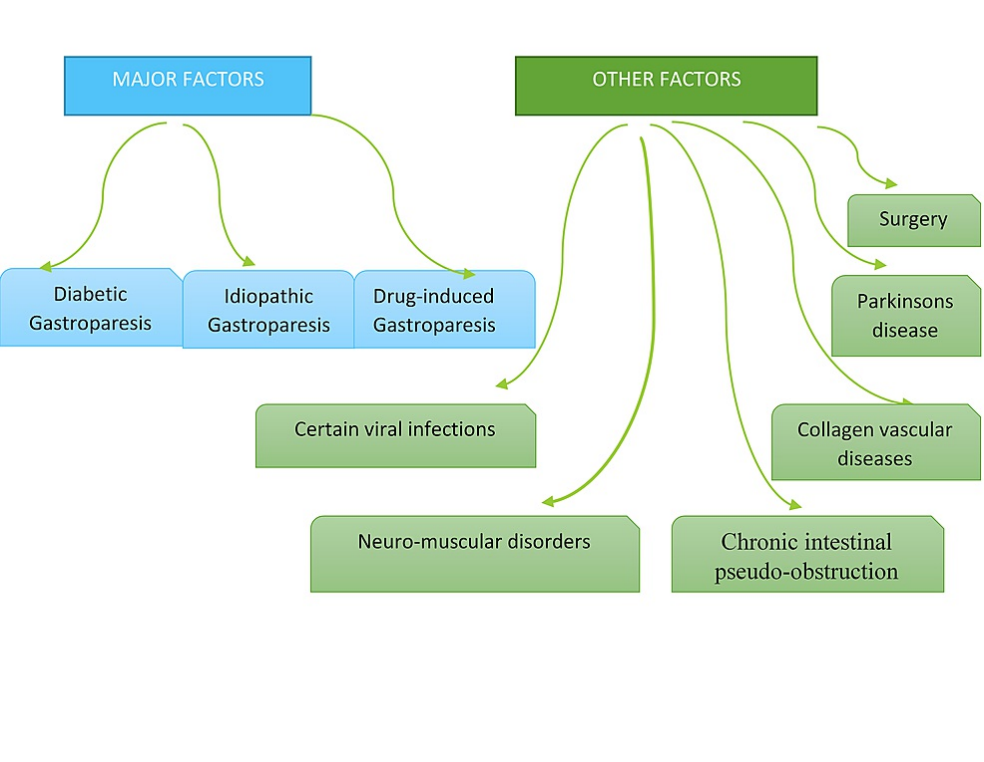
Risk factors for gastroparesis.

Various factors have been implicated in the etiology of gastroparesis, as represented in Figure 1. The risk factors significantly impact the severity of the presentation [26]. In a population-based study in the United Kingdom, idiopathic gastroparesis (39.4%) was the most frequent risk factor, followed by diabetes (37.5%) and drug-induced (19.6%) gastroparesis; all other etiologies were rare (<2%) [7]. This is comparable to the findings of Duffey et al. [26]. However, the prevalence of drug-induced gastroparesis in their study was higher (29.4%) [26]. Medications such as opioid analgesics, anticholinergics, and cannabinoids such as marijuana can affect GI motility [2]. Diabetes mellitus is a systemic disease most often associated with gastroparesis [9,27]. A higher proportion of type 2 diabetics with gastroparesis were overweight or obese compared to others with type 1 or idiopathic gastroparesis [7].

Gastroparesis is a feature of autonomic neuropathy in longstanding diabetes, and its prevalence is increasing proportionally with the prevalence of diabetes [13,14]. Hyperglycemia is a potential inhibitor of motilin which is known to stimulate GI contraction. A blood glucose level of more than 10 mmoI/L can induce electrogastric dysrhythmias and lower intragastric pressure resulting in delayed gastric emptying [28]. Conversely, diabetic gastroparesis also has an erratic impact on glycemic control. The role of surgery cannot be undermined. Postsurgical gastroparesis can occur in patients with vagus nerve injury during surgical procedures [14,28]. Rarely, certain viral infections can be associated with acute dysautonomia [6].

Additionally, other conditions such as collagen vascular diseases, Parkinson’s disease, and chronic intestinal pseudo­-obstruction have been implicated as predisposing factors to gastroparesis via a common pathophysiological mechanism of induction of neuromuscular dysfunction [6,29].

Further, gastroparesis has been associated with certain malignancies. Gallbladder malignancy is known to be associated with gastroparesis, with a majority presenting with anatomical obstruction of the gastric outlet or physiological abnormalities such as visceral or somatic neuropathy [30]. Ghoshal et al. reported a case of cholangiocarcinoma-associated gastroparesis attributed to paraneoplastic autonomic neuropathy [31].

Clinical presentation of gastroparesis

A wide range of GI symptoms is associated with gastroparesis including nausea, vomiting, early satiety, postprandial fullness, and abdominal pain [9,32,33]. Symptoms overlap with features of gastroesophageal reflux disease (GERD) and other motility disorders of the GI [34]. The delay in gastric emptying associated with gastroparesis can lead to the stimulation of transient lower esophageal sphincter relaxation, thereby worsening the symptoms of GERD [14]. Nausea and vomiting are well-recognized cardinal symptoms of gastroparesis [35]. However, abdominal pain and bloating are common and can be indicative of a disorder of gut perception or chronic pain syndrome rather than a motility problem [36]. This contradicts the study by Hasler et al. [37]. They reported that mildly severe bloating was found in the majority of gastroparesis patients (76%) and found increased use of antiemetics among patients with bloating [37]. As these symptoms progress, nutritional deficiencies and significant weight loss can be used as an index for the assessment of the severity of gastroparesis [13]. Anxiety disorders and depression have been associated with gastroparesis, with both negatively impacting the quality of life [38,39]. Patients reported feeling accused of malingering, which negatively impacted their social relationships, as well as changes to their sense of security and identity [40].

Diagnosis of gastroparesis

The majority of investigations in gastroparesis are designed to assess either gastric emptying or accommodation. Esophagogastroduodenoscopy is a frequently performed test. The presence of food in the stomach suggests ineffective antral motility [2]. The presence of alarming symptoms, such as weight loss, GI bleeding, anemia, or a family history of gastric cancer, suggests the recommendation of endoscopy or imaging studies to exclude mucosal or obstructive lesions [27] Occasionally, there can be major complications such as cardiopulmonary complications, infections, perforation, and bleeding [41].

Gastric emptying scintigraphy (GES) that entails the ingestion of a radiolabeled solid meal is considered the gold standard in the assessment of gastric emptying [42,43]. It is delayed if there is >60% retention at two hours and/or >10% retention at four hours [44]. Scintigraphy allows direct visualization of the test meal, thereby providing useful information regarding gastric activity. However, It is costly, not widely available, and exposes patients to radiation [44], necessitating the need for an alternative test.

Ultrasonography can be a cheap and valid alternative to scintigraphy [45]. It is readily available and does not involve radiation exposure. However, its limitations include dependence on user experience and the technical quality of the imaging [12,45]. The use of ultrasound to measure gastric emptying of solid meals can be achieved through cellulose-based gastric contrast agents which have a high correlation with scintigraphy [45].

Additionally, stable isotope breath tests and wireless motility capsules have been approved by the Food and Drug Administration (FDA) as non-invasive substitutes for scintigraphy [46]. The stable isotope breath test is easy to perform, is cost-effective, and associated with negligible exposure of patients to ionizing radiation [6]. However, it is limited by low‐calorie and low‐fat test meals which likely underestimate the prevalence of gastric emptying abnormalities [6,34]. There is also poor reproducibility of reliable results in patients with intestinal malabsorption or liver insufficiency [34].

Electrogastrography (EGG) is a non-invasive tool for the diagnosis of delayed gastric emptying. Transcutaneous EGG evaluates slow-wave activity and peak potentials of gastric contractions by measuring gastric myoelectrical activity [16,47]. Abnormalities in the postprandial EGG offer a better prediction of delayed gastric emptying [16]. Slow-wave frequency and rhythm were previously defined by cutaneous EGG [48]. However, their reliability is limited by the lack of spatial resolution, resulting in a need for a high-resolution (HR) electrical mapping [19,49].

The use of HR electrical mapping is well established in cardiac arrhythmias and is currently showing huge potential in the field of gastroenterology [4,49,50]. It involves recording electrical activity in a spatiotemporal manner [19,51]. In an animal study, Angeli et al. [52] reported that mucosal HR mapping was consistent with serosal mapping in frequency, propagation, and velocity, although its amplitude was reduced compared to serosal mapping. This has provided a foundation for future prospects of mucosal mapping with an emphasis on the improvement of mucosal signal quality [52]. The limitations to the progression of this field of research include cost, difficulty in electrode construction, and invasiveness of current technologies [51]. This problem has been largely solved by endoscopic implantable wireless devices which are revolutionary in the management of gastric dysrhythmias [53].

The assessment of gastric accommodation is valuable in the evaluation of a patient with gastroparesis. Gastric balloon barostat has been considered the gold standard for assessing impaired gastric accommodation [12,54]. However, it is invasive, uncomfortable to patients, and not widely available [12,55]. An inflatable balloon in the stomach is connected by a tube through the esophagus to an external air supply which ensures a constant pressure [5].

Single-photon emission computed tomography (SPECT), which is a non-invasive tool for estimating gastric accommodation, involves the injection of intravenous Tc-99m to radiolabel the gastric mucosa [55]. Although the use of SPECT imposes a limited radiation burden, it is yet to be approved for routine clinical use [12]. Table 1 lists the various diagnostic modalities for gastroparesis.

**Table 1:** Diagnostic modalities for gastroparesis. FDA: Food and Drug Administration

Investigations	Advantages	Disadvantages
Ultrasonography	Cheap, readily available, and no exposure to radiation	Dependence on operator experience and quality of the imaging. Measurement of gastric emptying of solids is challenging [14]
Gastric balloon barostat	The gold standard for the assessment of gastric accommodation [12,54]	It is invasive, uncomfortable to patients, and not widely available [12]
Single-photon emission computed tomography scan	Non-invasive estimate of gastric accommodation [12,55]	Unlike the gastric ballon barostat, it cannot measure gastric pressure [55]
Gastric emptying scintigraphy	It directly visualizes the test meal, thereby providing information regarding regional gastric activity	It is costly, not widely available, and exposes patients to radiation [44]
Stable isotope breath test	Approved by the FDA as a non-invasive substitute for scintigraphy [46]. It is easy to perform and cost-effective, with minimal exposure to ionizing radiation [6]	The use of low‐calorie, low‐fat test meals underestimates the prevalence of gastric emptying abnormalities in real life. Poor reliability in patients with intestinal malabsorption or liver insufficiency [34]
Esophagogastroduodenoscopy	It is useful in excluding organic diseases and can detect the presence of food in the stomach, suggesting ineffective antral motility [2]	It can be associated with some major complications such as cardiopulmonary complications, infections, perforation, and bleeding [41]
Electrogastrography	It evaluates slow-wave activity and peak potentials of the gastric contractions by measuring gastric myoelectrical activity [16,47]	Its reliability is limited by the lack of spatial resolution [19,49]
High-resolution electrical mapping	It permits the recording and reconstruction of patterns of electrical activation in spatiotemporal detail [21]	The high cost of multichannel acquisition systems, difficulty in electrode construction, and the high complexity and time-intensiveness of analytical tasks [51]

Recent advances in the management of gastroparesis

**Figure 2:**
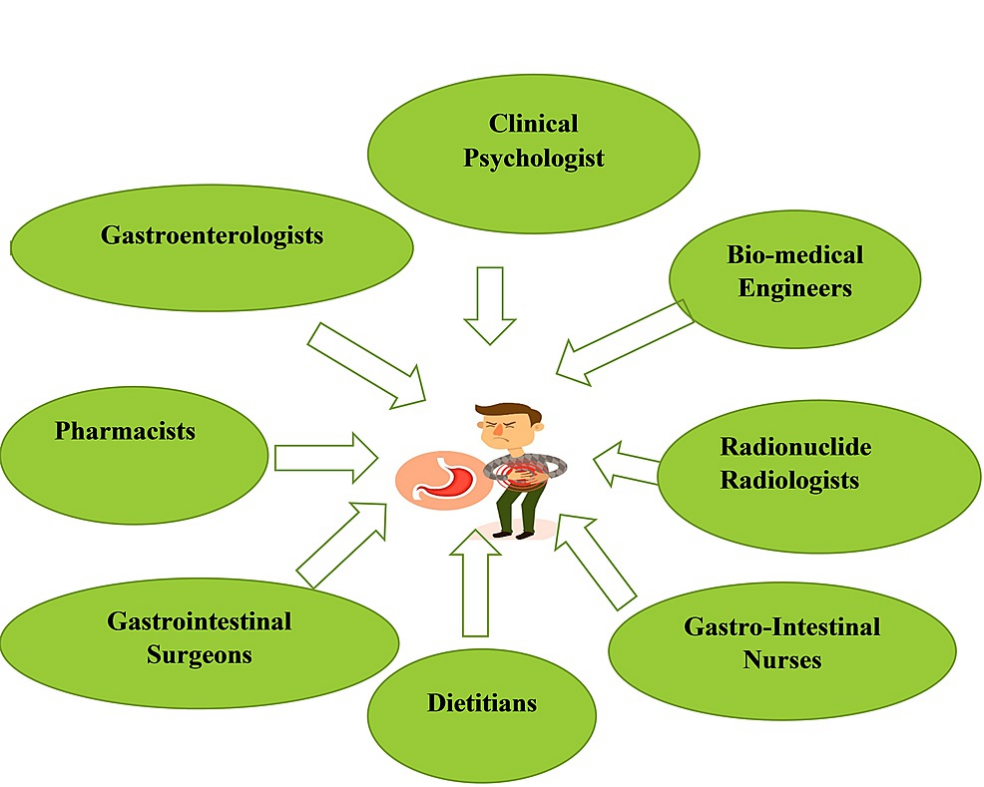
Multidisciplinary approach to gastroparesis management.

Although gastroparesis has been traditionally managed medically, when symptoms are refractory, it poses a therapeutic dilemma and may require surgical therapy [56]. Gastroenterologists work in multispecialty teams along with surgeons and radiologists to meet the needs of these patients [57]. In most countries, pharmacists and nurses are involved in monitoring the efficacy and adverse effects of drugs prescribed by doctors using the pharmacovigilance systems [58]. Therefore, a team effort is required for the effective management of patients, as illustrated in Figure 2.

Combination therapies targeting various pathogenetic mechanisms are theoretically appropriate and need to be further studied [59]. Although prokinetic agents, such as metoclopramide, erythromycin, domperidone, and cisapride, are commonly used, there is a lack of validated data and algorithms supporting most drugs [60]. This poses a major challenge to the physician, thereby imposing an enormous burden on the healthcare system [4,28]. Metoclopramide, a dopamine agonist with prokinetic and antiemetic activity, is the only medication approved by the FDA in the United States [59,61]. Synthetic analogs of motilin and ghrelin have been widely investigated for the treatment of gastroparesis. Erythromycin, a motilin-like molecule and macrolide antibiotic, enhances gastric emptying by inducing migrating motor complex contractions; however, its use is limited by adverse effects such as abdominal cramps, nausea, diarrhea, QT prolongation, and tachyphylaxis [4,59].

Novel pharmacological drugs for gastroparesis

Several novel pharmacologic agents are upcoming and promising for individualized therapy for patients with gastroparesis (Table 2). Relamorelin, a ghrelin receptor agonist, relieves pivotal symptoms such as vomiting, nausea, bloating, fullness, and pain in patients with diabetic gastroparesis by stimulating gastric emptying [4,62]. It also stimulates colonic contractions and can be useful for patients with chronic constipation [62]. Other ghrelin agonists that are under investigation include anamorelin, ibutamoren, and ipamorelin [62]. Prucalopride, a selective 5HT4 receptor agonist, has been effectively used to treat constipation-predominant gastroparesis [59]. This is supported by the study reported by Carbone et al. [63]. Aprepitant is a neurokinin-1 (NK-1) receptor antagonist used for postsurgical and cancer chemotherapy-induced nausea and vomiting. It inhibits NK-1 receptors that mediate the effects of substance P, which is a neurotransmitter involved in the perception of pain [64]. In a multicenter randomized trial, aprepitant did not significantly reduce the symptoms of nausea on the Visual Analog Scale; however, there was a reduction in symptom severity using a more common and validated measure for the secondary outcome (Gastroparesis Cardinal Symptom Index) [65]. Levosulpiride is the levorotatory enantiomer of sulpiride whose pro-kinetic effect involves the inhibition of enteric D2 and serotoninergic 5HT4 receptor agonist effect [66,67]. It is associated with mood elevation and may show promising results in a subset of gastroparesis patients with co-existing anxiety disorders. However, its use in gastroparesis can be complicated by parkinsonian features whose occurrence is independent of treatment duration [67]. Mirtazapine is one of the medications that target the gut-brain axis, thereby ameliorating the symptoms of gastroparesis [68,69].

**Table 2:** Novel pharmacological drugs for gastroparesis.

Drugs	Mechanism of action
Relamorelin, anamorelin, ibutamoren, ipamorelin	Ghrelin receptor agonist that has been shown to accelerate gastric emptying [4,62]
Prucalopride	Selective 5HT4 receptor agonist used in the treatment of constipation. It has been effectively used to enhance the gastric emptying rate in patients with gastroparesis [59]
Aprepitant	Neurokinin-1 receptor antagonist which inhibits the effects of the excitatory neurotransmitter substance P [64]
Levosulpiride	The levorotatory enantiomer of sulpiride whose pro-kinetic effect is mediated through the blockade of enteric inhibitory dopaminergic type 2 (D_2_) and serotoninergic 5HT4 receptor agonist effect [66,67]
Mirtazapine	A tetracyclic antidepressant with 5-HT1a receptor agonist activity in the central and peripheral nervous system [68,69]

Co-existing anxiety or depression correlates with the findings in other functional GI disorders and provides the rationale for alternative therapies targeting the brain. Gastroparesis patients require treatment that goes beyond the conventional approach [40]. A comprehensive mental intervention improved the postsurgical recovery time of gastroparesis patients [70]. Therefore, mental health units are encouraged to foster a patient-centered psychological support program. Cognitive-behavioral therapy has shown prospects in certain functional GI diseases [71], but its role in the management of gastroparesis needs to be further investigated.

In cases of refractory gastroparesis, surgical interventions have been useful in improving patients’ quality of life [11,56]. However, most surgical procedures are invasive and can be associated with debilitating complications. To reduce the burden of these complications, several endoscopic procedures are emerging and may show future prospects in the management of gastroparesis.

Pyloric therapies such as botulinum toxin injection, stent placement, pyloroplasty, and pyloromyotomy therapeutically reduce the pressure gradient across the pyloric sphincter, thereby increasing gastric emptying [72]. Fundoplication at the time of pyloroplasty is often an important adjunct in patients with co-existing GERD [72]. These interventions have promising outcomes and will likely serve as the basis for further research. Botulinum toxin A inhibits acetylcholine release at the neuromuscular junction [53] and impairs neuromuscular conduction, causing transient muscle paralysis [20,73]. In a retrospective analysis, symptom improvement was reported by 35% of the patients [73]. However, neither scintigraphy nor manometric parameters could predict treatment outcomes after botulinum toxin injection [73]. Non-pyloric therapies, such as venting gastrostomy and gastric electrical stimulation (GES), have been reported to improve symptoms [11]. Although the field of gastric electrophysiology is evolving, it is constrained by the complexity and poor understanding of the electrophysiology of the stomach [5].

Biomedical engineers are key players in supporting the knowledge of clinical professionals in the prevention, diagnosis, and treatment of diseases, as well as in modifying the anatomy of the human body with emerging devices. They need to ensure that the devices are safe, effective, and perform optimally [74]. GES is one of the innovative measures proposed as a salvage therapy for medically refractory gastroparesis [75,76]. Electrical waves, long-pulse or low-frequency stimulation, can be deployed to enhance gastric emptying. However, high power consumption is a limitation in its clinical use. The second type of GES referred to as short-pulse duration or high-frequency-low-energy stimulation is more suitable for clinical use. McCallum et al. found that patients who had GES for six weeks had rapid symptom reduction that was sustained after three months of device withdrawal [76]. This report is consistent with that by Angeli et al., although there was no immediate alteration of slow-wave velocity, amplitude, or frequency [77]. These therapies can be associated with various side effects including, paresthesia, lead migration/dislodgement, or migration of neurostimulator. Paresthesia was effectively resolved by device re-programming [76]. Moreover, mapping in conscious patients is associated with major technical issues that can be overcome by technological advancements, of which wireless transmission is promising.

The role of the nutritionist must not be neglected in managing this challenge. In patients with diabetic gastroparesis, diet modifications are pivotal in symptom reduction and glycemic control [74]. Nutritional assessment should be initially done, and dietary interventions should be based on the patient’s nutritional status, upper GI symptoms, and dietary habits [74]. There should be increased intake of liquid nutrients as liquid emptying is often preserved, while fat and fiber diet which slows gastric emptying should be minimized [4]. Smoking and alcohol should also be avoided [11,27].

## Conclusions

There is an urgent need for more interdisciplinary collaboration to target various pathophysiological mechanisms implicated in gastroparesis. Researchers should also develop robust theoretical frameworks and study designs for clinical trials in gastroparesis for developing individualized therapy. This could be the solution to the long-awaited breakthrough in the management of gastroparesis and should be embraced by various specialties.
